# Exploring water radiolysis in proton cancer therapy: Time-dependent, non-adiabatic simulations of H^+^ + (H_2_O)_1-6_

**DOI:** 10.1371/journal.pone.0174456

**Published:** 2017-04-04

**Authors:** Austin J. Privett, Erico S. Teixeira, Christopher Stopera, Jorge A. Morales

**Affiliations:** Department of Chemistry and Biochemistry, Texas Tech University, Lubbock, Texas, United States of America; Syddansk Universitet, DENMARK

## Abstract

To elucidate microscopic details of proton cancer therapy (PCT), we apply the simplest-level electron nuclear dynamics (SLEND) method to H^+^ + (H_2_O)_1-6_ at E_*Lab*_ = 100 keV. These systems are computationally tractable prototypes to simulate water radiolysis reactions—i.e. the PCT processes that generate the DNA-damaging species against cancerous cells. To capture incipient bulk-water effects, ten (H_2_O)_1-6_ isomers are considered, ranging from quasi-planar/multiplanar (H_2_O)_1-6_ to “smallest-drop” prism and cage (H_2_O)_6_ structures. SLEND is a time-dependent, variational, non-adiabatic and direct method that adopts a nuclear classical-mechanics description and an electronic single-determinantal wavefunction in the Thouless representation. Short-time SLEND/6-31G* (*n* = 1–6) and /6-31G** *(n* = 1–5) simulations render cluster-to-projectile 1-electron-transfer (1-ET) total integral cross sections (ICSs) and 1-ET probabilities. In absolute quantitative terms, SLEND/6-31G* 1-ET ICS compares satisfactorily with alternative experimental and theoretical results only available for *n* = 1 and exhibits almost the same accuracy of the best alternative theoretical result. SLEND/6-31G** overestimates 1-ET ICS for *n* = 1, but a comparable overestimation is also observed with another theoretical method. An investigation on H^+^ + H indicates that electron direct ionization (DI) becomes significant with the large virtual-space quasi-continuum in large basis sets; thus, SLEND/6-31G** 1-ET ICS is overestimated by DI contributions. The solution to this problem is discussed. In relative quantitative terms, both SLEND/6-31* and /6-31G** 1-ET ICSs precisely fit into physically justified scaling formulae as a function of the cluster size; this indicates SLEND’s suitability for predicting properties of water clusters with varying size. Long-time SLEND/6-31G* (*n* = 1–4) simulations predict the formation of the DNA-damaging radicals H, OH, O and H_3_O. While “smallest-drop” isomers are included, no early manifestations of bulk water PCT properties are observed and simulations with larger water clusters will be needed to capture those effects. This study is the largest SLEND investigation on water radiolysis to date.

## Introduction and background

Proton cancer therapy (PCT) is an approved type of radiotherapy that utilizes high-energy H^+^ projectiles to fight cancer [1–7]. The ultimate effect of this radiation is to damage the DNA of cancerous cells [1–7]. If left unrepaired, this damage produces various anomalies in cancerous cells that eventually lead to their death (apoptosis) [1–7]. While PCT damages both healthy and cancerous cells, the latter have a high rate of division and a reduced ability to repair damaged DNA. Thus, cancer cells are particular vulnerable to radiation attacks[8].

PCT radiation is applied as collimated beams of H^+^ projectiles at an initial kinetic energy of 70–250 MeV [1–7]. As they penetrate the patient’s body, these projectiles lose their energy through molecular interactions until they reach thermal energy in deep tissues. The radiation energy deposited in the tissues is a measure of its potential for DNA damage. Typically, a plot of the radiation energy loss *vs*. the radiation travelled distance exhibits a maximum of energy deposition [2]. Conventional X-ray therapy exhibits a broad deposition maximum not far after the photons’ penetration into the body, followed by a gradual energy loss at deeper penetrations [2]. In contrast, PCT (and other ion therapies) exhibits a sharp maximum peak known as the Bragg peak; that peak occurs just before the H^+^ projectiles are stopped in deep tissues [2]. Thus, by focusing Bragg peaks on a deep tumor, PCT inflicts a maximum DNA damage on that region and a minimum DNA damage on the surrounding healthy tissues [1–7].

In all radiotherapies, the radiation predominantly interacts with cellular H_2_O because the latter constitutes about 70% of the human cell mass. This interaction triggers water radiolysis reactions—i.e. a series of cascade hydrolytic reactions producing DNA-damaging species. In PCT, water radiolysis generates various secondary species: (a) free radicals (e.g. H^+^ + H_2_O → H^+^ + H·+ OH·)[2–4, 6, 9], (b) secondary ions (e.g. H^+^ + H_2_O → 2H^+^ *+* OH^-^)[2–4, 6, 9], (c) reactive molecules (e.g. H^+^ + 2H_2_O → H^+^ + H_2_ + H_2_O_2_) [10], and (d) localized heat in the medium [2–4, 6, 9]. These species can react with H_2_O and generate similar tertiary species and so forth. All these reactive products eventually reach cellular DNA and cause various types of damage: DNA bases’ fragmentations and deletions, and single-, double- and clustered-strand DNA breaks [2–5, 11].

PCT comprises various processes spanning different space (*l* = 10^−10^–10^0^ m) and time (*t* = 10−21–10^5^ s) scales [2–4]. For instance, water radiolysis reactions, DNA damage at the genome level [12] and tumor remissions lie at the microscopic (10^−10^ ≤*l*≤10^−8^ m), mesoscopic (10^−8^ ≤*l*≤10^−3^ m) and macroscopic (10^−3^ ≤*l*≤10^0^ m) scales, respectively. The scale of a process determines the appropriate methods for its study. Thus, in theoretical/computational studies, microscopic water radiolysis reactions can be feasibly simulated with *ab initio* quantum-mechanics methods. In contrast, mesoscopic energy-loss and Bragg peak processes are only tractable with Monte Carlo (MC) models [12–14]. Quantum-mechanics and MC methods act in synergy: the former predict properties (e.g. reaction cross sections) required as input data for MC simulations [9, 12–20], and the latter calculate proper radiation doses for treatments [18].

Although PCT is clinically approved, various PCT details at the microscopic scale remain uncertain [1–7]. Knowledge of those details is essential for a rational design of PCT seeking to maximize its therapeutic power and minimize its side effects [1–7, 18]. While the predominant paradigm for cancer research is experimental/clinical, theoretical/computational methods can reveal microscopic details of PCT more exhaustively than experimental/clinical techniques and without putting human subjects at risk. Therefore, time-independent scattering [21–23] and time-dependent dynamics methods [6, 24–27] have been applied to computationally feasible prototypes of PCT reactions (e.g. H^+^ + (H_2_O)_1–4_ to model water radiolysis reactions [6, 21–26, 28] and H^+^ + DNA/RNA bases to model DNA proton damage [6, 21, 22, 26, 27]).

Among quantum-mechanics methods for PCT [6, 24–27], the electron nuclear dynamics (END) theory [26, 29] offers distinctive capabilities to study PCT reactions. END is a (1) time-dependent, (2) direct and (3) non-adiabatic method to simulate chemical reactions. These attributes are valuable for PCT simulations because they afford: (1) time-dependent detail, (2) independence from predetermined potential energy surfaces, and (3) capability of describing high-energy non-adiabatic processes [e.g. electron transfers (ETs)]. Among different END versions [26, 29, 30], the simplest-level (SL) END (SLEND) describes the nuclei and electrons in terms of classical mechanics and a Thouless single-determinantal wavefunction, respectively [26, 29, 31]. Thus, SLEND possesses a suitable balance between accuracy and computational feasibility to simulate large PCT prototypes (cf. previous SLEND studies of H^+^ + (H_2_O)_*n*_, *n* = 1 [24, 28], 2 [25], and 3–4 [6, 26] and of H^+^ + DNA/RNA bases and DNA base pairs [6, 26, 27]).

**Fig 1:**
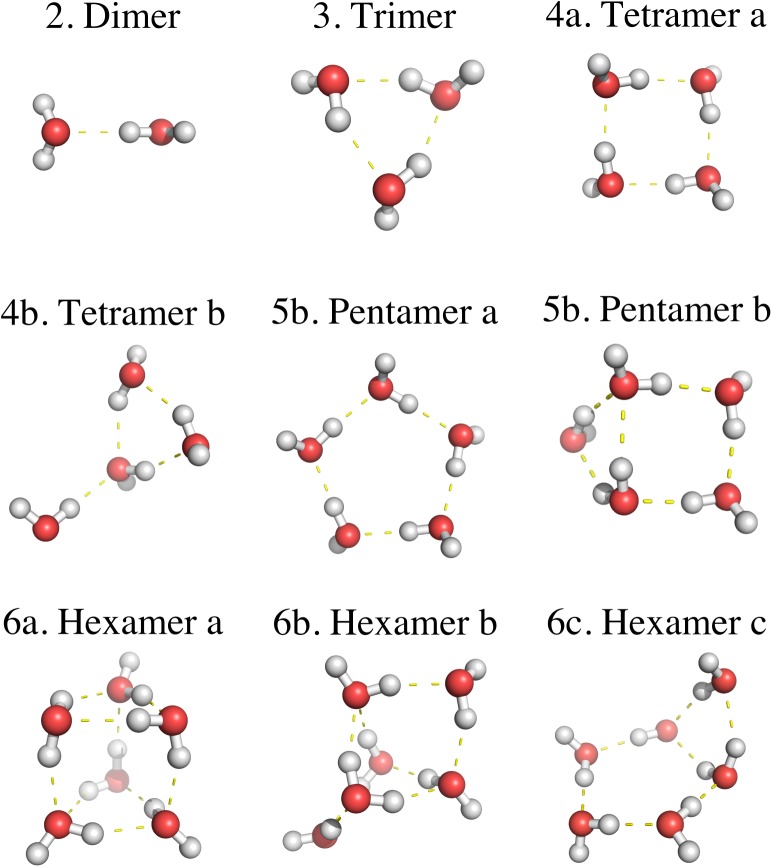
UHF/6-31G** [(H_2_O)_2-5_] and UHF/6-31G*[(H_2_O)_6_] optimized geometries of the water cluster isomers selected for this study. When two or more isomers of a given water cluster are considered, they are depicted/listed in the order of increasing energy; the first depicted/listed isomer is the lowest-energy isomer in its whole known series with the present theory [e.g. 6a is the lowest-energy (H_2_O)_6_ structure out of 12 known (H_2_O)_6_ isomers[34]]. 6a and 6b are the prism and cage isomers of (H_2_O)_6._

Following the aforesaid precedents [6, 21–26, 28], we present herein an exploratory SLEND study of PCT water radiolysis reactions with the H^+^ + (H_2_O)_1-6_ prototypes at *E*_*Lab*_ = 100 keV. This energy is selected because it corresponds to the average Bragg peak energy in bulk water [32], the medium where water radiolysis occurs. However, no *ab initio* quantum-mechanics methods can simulate bulk water due to prohibitive computational cost and, therefore, those methods treat the above-mentioned water-clusters prototypes. Surprisingly, most quantum-mechanics studies of water radiolysis have utilized the smallest prototype: H^+^ + H_2_O [21–24, 28], whose “cluster” is the farthest from being bulk water. While these H^+^ + H_2_O studies were indeed useful for investigating radiolysis processes [21–24, 28], they could not completely capture the processes occurring in bulk water. For that reason, previous SLEND studies explored H^+^ + (H_2_O)_*n*_ prototypes with n = 2 [25] and 3–4 [6, 26]. However, these studies still concentrated on the smallest possible clusters and involved a limited number of proton-cluster orientations and simulations. Therefore, to overcome all the discussed limitations, we study herein the H^+^ + (H_2_O)_1-6_ prototypes that include ten isomers in a larger series of clusters (H_2_O)_1-6_ (cf. Fig 1) and involve a larger number of proton-cluster orientations (60) and simulations (25,020).

The selected (H_2_O)_1-6_ series contains the initial terms of the long progression from molecular H_2_O to bulk water. Specifically, the first terms in this progression, (H_2_O)_1-5,_ have mono- and di-cyclic quasi-planar/multiplanar structures [33–35] exhibiting no “bulky” shapes (cf. Fig 1), but two isomers in the last term—the prism and cage isomers of (H_2_O)_6_—have multi-cyclic, three-dimensional structures exhibiting drop-like shapes [34, 35] (cf. Fig 1). In fact, these two (H_2_O)_6_ isomers, particularly the prism, are considered the smallest possible drops of water [34, 35]—i.e. the minimum water structures that manifest the three-dimensional hydrogen-bond structure [35] and solubility properties [36, 37] of bulk water. Based on those facts, we expected that this series would reveal the earliest manifestations of bulk water effects on PCT properties; however, the present results do not display such manifestations and suggest that even larger water clusters should be considered (cf. Results and Discussion Section). Despite that outcome, all the predicted properties and reactions of H^+^ + (H_2_O)_2-6_ have never been measured or calculated before; therefore, the present results are truly predictive and fill a gap in the medical physics literature [16]. Furthermore, these results are important to understand PCT more thoroughly and to model water radiolysis processes [15–20] and radiation dosages [12–14, 16–20] with MC methods. Finally, it should be noticed that our simulated phenomena are the first processes occurring upon a short-time direct collision of a proton with moderate size clusters. Other post-collision phenomena contributing to PCT such as local temperature increases [2–4] and hypothesized shock waves [38] in water require for their modelling longer simulation times, larger clusters and even different theoretical methods; therefore, those phenomena are not reproduced by the current simulations.

## Methodology

The END theory and its SLEND version have been reviewed in detail in Refs. [26, 29, 39]; therefore, we provide a brief outline of them. END is a variational, time-dependent, direct, and non-adiabatic dynamical method [26, 29]. END prescribes a total trial wavefunction $\left|\Psi_{Total}^{END}\right\rangle =\left|\Psi_{N}^{END}\right\rangle \;\left|\Psi_{e}^{END}\right\rangle$, which consists of nuclear $\left|\Psi_{N}^{END}\right\rangle$ and electronic $\left|\Psi_{e}^{END}\right\rangle$ wavefunctions, and treats $\left|\Psi_{Total}^{END}\right\rangle$ under the time-dependent variational principle (TDVP) [40]. The various versions of END differ in the kind of descriptions for the nuclei and electrons (e.g., density functional theory [30] [26] for electrons). In SLEND, the nuclear wavefunction $\left|\Psi_{N}^{SLEND}\right\rangle$ for a system having *N*_*N*_ nuclei is the product of 3*N*_*N*_ frozen, narrow Gaussian wave packets:

$$\langle X\left|\Psi_{N}^{SLEND}\left(t\right)\right\rangle =\langle X\left|R\left(t\right),\;P\left(t\right)\right\rangle =\prod_{A=1}^{3N_{N}}\exp\left\{-{\left[\frac{X_{A}-R_{A}\left(t\right)}{2\Delta R_{A}}\right]}^{2}+iP_{A}\left(t\right)\left[X_{A}-R_{A}\left(t\right)\right]\right\}$$ (1)

with average positions **R**_*A*_(*t*), average momenta **P**_*A*_(*t*) and widths Δ*R*_*A*_. To lower computational cost, the zero-width limit, Δ*R*_*A*_ → 0, is applied to the nuclear wave packets after constructing the SLEND quantum Lagrangian (cf. next paragraph). That procedure generates a classical nuclear dynamics but with full retention of the nucleus-electron non-adiabatic coupling terms (cf. Eq 5). As proven previously [6, 26, 27], classical nuclear dynamics does not impair the accuracy of PCT simulations because they happen at high collision energies. The SLEND electronic wavefunction $\left|\Psi_{e}^{SLEND}\right\rangle$ is a spin-unrestricted, single-determinantal wavefunction in the Thouless representation [31]. Specifically, taking *N*_*e*_ occupied {*ψ*_*h*_}, and *K* – *N*_*e*_ virtual {*ψ*_*p*_} molecular spin-orbitals (MSOs), $\left|\Psi_{e}^{SLEND}\right\rangle$ is [31]:

$$\left|\Psi_{e}^{SLEND}\right\rangle =\left|z\left(t\right);R\left(t\right),P\left(t\right)\right\rangle =\det\left\{\chi_{h}\left[x_{h};\;z\left(t\right),R\left(t\right),P\left(t\right)\right]\right\}=\text{exp}\left[\sum_{h=1,\;p=N_{e}+1}^{N_{e},\;K}z_{ph}\left(t\right)b_{p}^{†}b_{h}\right]\left|0\right\rangle ;$$ (2)

where $\left|0\right\rangle =|\psi_{N_{e}}\ldots \psi_{1}\rangle$ is an unrestricted Hartree-Fock (UHF) reference state and {*χ*_*h*_} are dynamical spin-orbitals (DSOs)

$$\chi_{h}\left[x;\;z\left(t\right),R\left(t\right),P\left(t\right)\right]=\psi_{h}\left[x;\;R\left(t\right),P\left(t\right)\right]+\sum_{p=N_{e}+1}^{K}z_{ph}\left(t\right)\psi_{p}\left[x;\;R\left(t\right),P\left(t\right)\right];\;\left(1\leq h\leq N_{e}\right)$$ (3)

with complex-valued molecular coefficients {*z*_*ph*_(*t*)}. The MSOs are obtained at initial time at the UHF level. The MSOs are constructed with travelling atomic basis set functions $\phi_{A_{i}}\left(r_{i};R_{A},P_{A}\right)$—i.e., Slater-type orbitals in terms of contracted Gaussian-type orbitals on the moving nuclear centers **R**_*A*_(*t*) and augmented with electron translation factors (ETFs) [41] to include explicit nuclear momenta **P**_*A*_(*t*) effects. MSOs and DSOs are spin-unrestricted; therefore, the unrestricted determinant |**z**(*t*);**R**(*t*),**P**(*t*)⟩ can reasonably describe bond-breaking/-forming processes. The Thouless representation provides a non-redundant and singularity-free parameterization of an evolving single-determinantal state [26, 29, 40].

The SLEND dynamical equations are obtained according to the TDVP[40]. First, the quantum Lagrangian [26, 29, 40] $L_{SLEND}=\left\langle \Psi_{Total}^{SLEND}\;\right|\;\left(i/2\right)\;(\vec{\partial }/\partial t-\overset{\leftarrow }{\partial }/\partial t)-{\hat{H}}_{Total}\;\left|\;\Psi_{Total}^{SLEND}\right\rangle /\left\langle \Psi_{Total}^{SLEND}\;\right|\;{\Psi_{Total}^{SLEND}\rangle }^{-1}$ is constructed and then the zero-width limit is applied to the nuclear wave packets. Subsequently, the stationary condition is imposed to the quantum action [26, 29, 40] *A*_*SLEND*_, $\delta A_{SLEND}=\delta \int_{t_{1}}^{t_{2}}L_{SLEND}(t)dt=0$. The resulting SLEND dynamical equations are [26, 29]:

$$\left[\begin{array}{cccc} iC & 0 & iC_{R} & iC_{P}\\ 0 & -iC^{*} & -iC_{R}^{*} & -iC_{P}^{*}\\ iC_{R}^{†} & -iC_{R}^{T} & C_{RR} & -I+C_{RP}\\ iC_{P}^{†} & -iC_{P}^{T} & I+C_{PR} & C_{RP} \end{array}\right]\left[\begin{array}{c} \frac{dz}{dt}\\ \frac{dz^{*}}{dt}\\ \frac{dR}{dt}\\ \frac{dP}{dt} \end{array}\right]=\left[\begin{array}{c} \frac{\partial E_{Total}}{\partial z^{*}}\\ \frac{\partial E_{Total}}{\partial z^{}}\\ \frac{\partial E_{Total}}{\partial R}\\ \frac{\partial E_{Total}}{\partial P} \end{array}\right]$$ (4)

where *E*_*total*_ is the total (nuclear and electronic) energy and the dynamic metric matrices are [26, 29]

(CXY)ik,jl=−2Im∂2lnS∂Xik'∂Yjl|R′=RP'=P;(CXik)ph=∂2lnS∂zph*∂Xik|R′=RP'=P;Cph,qg=∂2lnS∂zph*∂zqg|R′=RP'=P (5)

where *X*_*ik*_ and *Y*_*jl*_ denote either **R**_*A* = *i*,*k*_ or **P**_*A* = *j*,*l*_ and *S* = ⟨**z**(*t*), **R**′(*t*), **P**′(*t*)|**z**(*t*), **R**(*t*), **P**(*t*)⟩. **C**_**R**_ and **C**_**RR**_ are equivalent to the ordinary non-adiabatic coupling terms [42]. Neglect of these terms seriously impairs the accuracy of the SLEND non-adiabatic dynamics [43]; therefore, those terms are kept in the present calculations. However, to lower computational cost, ETFs are not included in the present basis set so that **C**_**P**_ = **C**_**PR**_ = 0. The accelerated SLEND equations thus obtained have been successfully applied to various reactive and non-adiabatic processes, from a few eV[44] to the keV regime[45–48] and up to 900 keV [48] (cf. also the seminal END Ref. [29], especially page 948, where this approximation is applied exhaustively to high-energy non-adiabatic processes). At initial time, the reactants are prepared with positions $\left\{R_{A}^{i}\right\}$, momenta $\left\{P_{A}^{i}\right\}$ and Thouless electronic state |**z**^(*i*)^ = **0**, **R**^(*i*)^⟩ = |0⟩, i.e. the UHF ground state of the reactants’ super-molecule. When the reaction accesses the non-adiabatic regime, **z**(*t*) ≠ **0** and |**z**(*t*),**R**(*t*)⟩ becomes a superposition of ground |0⟩ and excited |… *h* → *p* …⟩ UHF states (cf. Eq 2)

$$\left|\Psi_{e}^{SLEND}\left(t\right)\right\rangle =\left|z(t);R\left(t\right),P\left(t\right)\right\rangle =\sum_{h=1,\;p=N_{e}+1}^{K,\;N_{e}+1}z_{ph}\left(t\right)\left|\ldots \;h\to p\;\ldots \right\rangle +\;\ldots$$ (6)

## Computational details

### Software

All the present SLEND simulations were computed with our END program PACE (Python-Accelerated Coherent states Electron-nuclear dynamics, T. V. Grimes, E. S. Teixeira and J. A. Morales, Texas Tech University, 2010–2016; cf. Ref. [26], Sect. 4). PACE combines several advanced computer science techniques such as a mixed programming language (Python for logic flow and Fortran and C++ for calculations), intra- and internode parallelization, and the fast OED/ERD atomic integral package [49] from the ACES III/IV [50] program. In addition, the water clusters’ geometries at initial time were computed with the NWChem [51] and GAMESS [52] programs.

### Water cluster structures at the initial states

**Fig 2:**
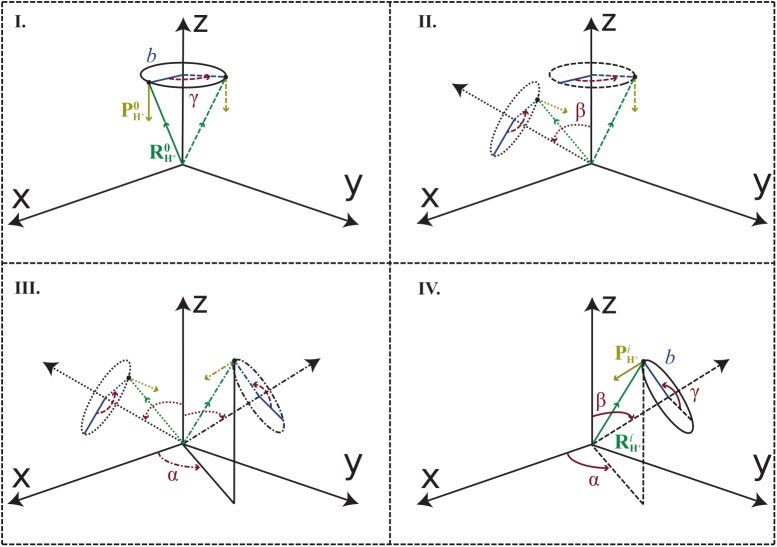
H^+^ + (H_2_O)_1-6_ initial conditions. A given water cluster (not depicted for clarity’s sake) is placed at rest with its center of mass at the origin of the coordinate axes and with its major (pseudo-)plane of symmetry with maximum coincidence with the x-y plane. The H^+^ projectile is initially prepared with position and momentum $R_{{\text{H}}^{+}}^{0}$ and $P_{{\text{H}}^{+}}^{0}$ and with impact parameter *b* (Panel I). Different projectile-target relative orientations Ω = (*α*, *β*, *γ*) are generated by rotating $R_{{\text{H}}^{+}}^{0}$ and $P_{{\text{H}}^{+}}^{0}$ through the *extrinsic* Euler angles *γ* (Panel I), *β* (Panel II), and *α* (Panel III) around the *space-fixed* z, y, and z axes, respectively. The definite initial conditions of the H^+^ projectile to start the simulations, $R_{{\text{H}}^{+}}^{i}$ and $P_{{\text{H}}^{+}}^{i}$, are shown in Panel IV (cf. text for more details).

Present SLEND simulations start with the super-molecular systems H^+^ + (H_2_O)_1-6_ optimized at the UHF level and having projectile-to-target [H^+^-to-(H_2_O)_1-6_] separations ≥ 30.00 a.u. (cf. Fig 2). Integration of the SLEND Eq 4 requires the evaluation of their various terms at numerous adaptive time steps on a total of 25,020 trajectories. Thus, for feasibility’s sake, the medium size basis sets 6-31G* [53] [for H^+^ + (H_2_O)_1-6_] and 6-31G** [53] [for H^+^ + (H_2_O)_1-5_] are adopted. These basis sets provide good water clusters’ geometries and energies (cf. Refs. [33, 34]; for these basis sets’ dynamical performance, cf. Electron Transfer Properties and Further Analysis Sub-Sections).

Numerous theoretical [33, 34, 54–63] and experimental [35, 64–66] studies have been devoted to determine the geometries and energies of water clusters because these systems are prototypes to study the structural [35] and solubility properties [36, 37] of bulk water. In fact, the scientific literature about water clusters is vast and growing. Therefore, for brevity’s sake, we limit ourselves to cite herein only the water clusters studies closely related to this investigation. It is well-known that the (H_2_O)_2-3_ clusters present one isomer each [34, 55]; however, the (H_2_O)_*n*_, *n* ≥ 4, clusters present a variable number of isomers that rapidly increase with the cluster size *n* [34, 55]. (H_2_O)_1-5_ present mono- and di-cyclic quasi-planar/multiplanar structures [33–35], whereas (H_2_O)_*n*_, *n* ≥ 6, present multi-cyclic, three-dimensional structures in addition to quasi-planar/multiplanar ones [34, 35] (cf. Fig 1). The three-dimensional (H_2_O)_6_ isomers (e.g. the prism and cage isomers named hexamer a and b in Fig 1) are considered the smallest drops of water [34, 35]. In general, theory and experiments agree in regard to the structures and relative energies of the (H_2_O)_1-5_ isomers, but discrepancies arise in the energy orders of the (H_2_O)_*n*_, *n* ≥ 6, isomers [35, 56]. Recent spectroscopy experiments have identified the cage isomer as the lowest-energy (H_2_O)_6_ structure [35, 66]. However, *ab initio* calculations at various levels of accuracy have disagreed on whether the prism [34, 57–59], the cage [60, 61], both of them [56], or the chair [62] isomer(s) is(are) the lowest-energy (H_2_O)_6_ structure(s). Ultimately, the most accurate calculations with the coupled-cluster with singles, doubles and perturbative triples [CCSD(T)] method have identified the prism isomer as the lowest-energy (H_2_O)_6_ structure [58].

For this investigation, we selected ten representative isomers in the (H_2_O)_1-6_ series: each single isomer of (H_2_O)_1-3,_ the two isomers of (H_2_O)_4,_ two isomers of (H_2_O)_5_ out of a total of at least four [34]_,_ and three isomers of (H_2_O)_6_ out of a total of at least twelve [34] (cf. Fig 1). The isomers were calculated at the UHF/6-31G* [(H_2_O)_1-6_] and /6-31G** [(H_2_O)_1-5_] levels. When two or more isomers of a (H_2_O)_n_ cluster are considered, they are depicted (cf. Fig 1) and listed (cf. Table 1) in their increasing order of energies [e.g. hexamer a (prism), hexamer b (cage), and hexamer c since $E_{\text{Hexamer b}}^{\text{Cage}}-E_{\text{Hexamer a}}^{\text{Prism}}$ = 4.2 kJ/mol and $E_{\text{Hexamer c}}^{}-E_{\text{Hexamer a}}^{\text{Prism}}$ = 15.4 kJ/mol with UHF/6-31G*). Notice that the UHF prism isomer is the lowest-energy (H_2_O)_6_ structure as predicted by CCSD(T) [58]. The first depicted/listed isomer is also the lowest-energy structure in its whole set of isomers [34]. The present UHF calculations of (H_2_O)_1-6_ are not intended to contribute to the resolution of the discrepancies about the (H_2_O)_6_ energies because these calculations do not attain the accuracy of CCSD(T) [58, 59]. Instead, these optimizations provide a good description of the water clusters [33, 34] for subsequent SLEND/6-31G* and /6-31G** simulations.

**Table 1:** Grids for the projectile impact parameter *b* for the SLEND simulations. [*b*]_1_ = grids for short-time simulations to calculate 1-electron-transfer total integral cross sections; [*b*]_2_ = grids for long-time simulations to predict fragmentation processes. Grid data are given as [*b*_Min_, *b*_Max_, Δ*b*] ⇒ *b* = *b*_Min_, *b*_Min_ + Δ*b*, *b*_Min_ + 2Δ*b* … *b*_Max_. All units are in a.u.

Water Cluster	Basis Set	[*b*]_1_	[*b*]_2_
Monomer	6-31G*	[0.0, 10.0, 0.5]	[0.0, 7.0, 0.5]
Monomer	6-31G**	[0.0, 10.0, 0.5]	
Dimer	6-31G*	[0.0, 12.0, 0.5]	[0.0, 4.0, 0.5]
Dimer	6-31G**	[0.0, 12.0, 0.5]	
Trimer	6-31G*	[0.0, 12.0, 0.5] + [12.0, 13.0, 1.0]	[0.0, 4.0, 0.5]
Trimer	6-31G**	[0.0, 12.0, 0.5] + [12.0, 13.0, 1.0]	
Tetramer a	6-31G*	[0.0, 9.0, 0.5] + [10.0, 15.0, 1.0]	[0.0, 6.0, 0.5]
Tetramer a	6-31G**	[0.0, 12.0, 0.5] + [13.0, 15.0, 1.0]	
Tetramer b	6-31G*	[0.0, 9.0, 0.5] + [10.0, 15.0, 1.0]	
Tetramer b	6-31G**	[0.0, 9.0, 0.5] + [10.0, 15.0, 1.0]	
Pentamer a	6-31G*	[0.0, 12.0, 0.5] + [13.0, 15.0, 1.0]	
Pentamer a	6-31G**	[0.0, 12.0, 0.5] + [13.0, 15.0, 1.0]	
Pentamer b	6-31G*	[0.0, 9.0, 0.5] + [10.0, 15.0, 1.0]	
Pentamer b	6-31G**	[0.0, 9.0, 0.5] + [10.0, 15.0, 1.0]	
Hexamer (prism)	6-31G*	[0.0, 11.0, 0.5] + [12.0, 16.0, 1.0]	
Hexamer (cage)	6-31G*	[0.0, 11.0, 0.5] + [12.0, 16.0, 1.0]	
Hexamer c	6-31G*	[0.0, 9.0, 0.5] + [10.0, 15.0, 1.0]	

### Initial states preparation and simulation times

Once the water clusters are optimized at the UHF/6-31G* and /6-31G** levels, the super-molecular systems H^+^ + (H_2_O)_1-6_ are assembled for the initial conditions (cf. Fig 2). The (H_2_O)_1-6_ targets are prepared at rest in their equilibrium geometries with their centers of mass placed at the origin of the laboratory-frame coordinate axes; the (H_2_O)_1-6_ major (pseudo-)planes of symmetry are placed with maximum coincidence with the x-y plane. The H^+^ projectile is first prepared with position $R_{{\text{H}}^{+}}^{0}=(b\geq 0,\;0,\;+30\;\text{a}.\text{u}.)$ and momentum $P_{{\text{H}}^{+}}^{0}=(0,\;0,-p_{{\text{H}}^{+}}^{z})$, where *b* ≥ 0 is the projectile impact parameter measured from the (H_2_O)_1-6_ centers of mass, and $p_{{\text{H}}^{+}}^{z}$ corresponds to *E*_*Lab*_ = 100 keV (cf. Fig 2, panel I). Having set $R_{{\text{H}}^{+}}^{0}$ and $P_{{\text{H}}^{+}}^{0}$, various projectile-target relative orientations can be generated by rotating a (H_2_O)_1-6_ target according to ordinary Euler angles prescriptions [67]. However, such a procedure involves the electronic re-optimization of the (H_2_O)_1-6_ targets at each new orientation. Therefore, since the H^+^ bare ion requires no electronic optimization, we adopted the easier and equivalent procedure of keeping a (H_2_O)_1-6_ target fixed while rotating the H^+^ projectile around. The definite initial conditions of the H^+^ projectile $R_{{\text{H}}^{+}}^{i}$ and $P_{{\text{H}}^{+}}^{i}$ are therefore obtained by rotating $R_{{\text{H}}^{+}}^{0}$ and $P_{{\text{H}}^{+}}^{0}$ through the *extrinsic* Euler angles [67] in the order: 1^st^, 0^0^ ≤ *γ* < 360^0^, 2^nd^, 0^0^ ≤ *β* ≤ 180^0^, and 3^rd^, 0^0^ ≤ *α* < 360^0^, around the *space-fixed* z, y, and z axes [67], respectively (cf. Fig 2, panels I, II and III for each angle rotation). *α* and *γ* are measured from the +x axis employing the $R_{{\text{H}}^{+}}^{0}$ and $P_{{\text{H}}^{+}}^{0}$ projections on the x-y plane and *β* is measured directly from the +z axis (cf. Fig 2). These rotations define projectile-target relative orientations Ω_*i*_ = (*α*_*i*_, *β*_*i*_, *γ*_*i*_). The definite initial conditions of the H^+^ projectile for the simulations, $R_{{\text{H}}^{+}}^{i}$ and $P_{{\text{H}}^{+}}^{i}$, are shown in Fig 2, panel IV. *α* and *β* determine the direction of an axis of incidence whereby an incoming H^+^ trajectory runs parallel to that axis with a lateral separation *b* ≥ 0; *γ* is the polar angle of that trajectory around the axis of incidence. In all simulations, the selected values of the Euler angles correspond to a 60-point grid: Ω_*i*_, 1≤*i*≤60, developed in Ref. [68]. This grid displays a uniform sampling of the orientation space and provides a numerical quadrature that ensures the invariance of Euler-angle integrals under several rotation operations (e.g. Wigner D-matrices satisfy $\int_{0}^{2\pi }\int_{0}^{\pi }\int_{0}^{2\pi }D_{M^{\prime }M}^{J}\;\left(\alpha ,\;\beta ,\;\gamma \right)\;\sin\beta d\alpha \;d\beta d\gamma =0$ for 2 ≤ *J* ≤ 5). For a given orientation Ω_*i*_, *b* is varied according to the grids defined in Table 1. Simulations for the calculation of 1-ET ICSs utilize the grids denoted as [*b*]_1_ in Table 1 and run for a total time of 30.00 a.u. (0.7257 fs); this simulation time ensures that the final projectile-target separation is at least equal to the initial one (30.00 a.u.). Simulations for the prediction of fragmentations utilize the grids denoted as [*b*]_2_ in Table 1 and run for a total time of 1,000 a.u. (24.19 fs); this much longer simulation time permits the manifestation of post-collision fragmentations that have longer time scales than those of the 1-ET processes. The described initial conditions generate a total of 25,020 trajectories to complete the H^+^ + (H_2_O)_1-6_ study.

### Final states analysis and properties calculations

By the end of a simulation, various auxiliary codes in the PACE package identify and analyze the final products and calculate dynamical properties. The most important properties calculated herein are the cluster-to-proton 1-ET total ICSs, *σ*_1−*ET*_, corresponding to H^+^ + (H_2_O)_1–6_ → H + different cluster products[69]

$$\sigma_{1-ET}=\frac{1}{4\pi }\int_{0}^{\infty }\int_{0}^{2\pi }\int_{0}^{\pi }\int_{0}^{2\pi }b\;P_{1\text{-ET}}\;\left(\alpha ,\;\beta ,\;\gamma ,\;b\right)db\;\sin\beta \;d\alpha \;d\beta \;d\gamma$$ (7)

where *P*_1−*ET*_(*α*, *β*, *γ*, *b*) is the probability of a 1-ET process from a bound electronic state of the cluster to a bound electronic state of the projectile, henceforth named bound-to-bound 1-ET for brevity’s sake. Notice that for atom-atom collisions involving spherically symmetric potentials, *P*_1−ET_ (*α*, *β*, *γ*, *b*) → *P*_1−ET_ (*b*), and Eq (7) simplifies to the familiar expression: $\sigma_{1-\text{ET}}=2\pi \int_{0}^{\infty }b\;P_{1-\text{ET}}\left(b\right)\;db$ [42]. In the present systems, an outgoing projectile can in practice capture up to two electrons: H^+^ + A → H^1−*n*^ + A^−*n*^, 0 ≤ *n* ≤ 2, because the probability of forming unstable H^1−*n*^ with *n* ≥ 3 is negligible. Under these conditions, *P*_*n*−ET_ (*α*, *β*, *γ*, *b*), 0 ≤ *n* ≤ 2, are [69]

$$\begin{array}{l} P_{0-\text{ET}}\left(\alpha ,\;\beta ,\;\gamma ,\;b\right)=\left(1-N_{\alpha }\right)\left(1-N_{\beta }\right);\\ P_{1-\text{ET}}\left(\alpha ,\;\beta ,\;\gamma ,\;b\right)=N_{\alpha }\left(1-N_{\beta }\right)+N_{\beta }\left(1-N_{\alpha }\right);\\ P_{2-\text{ET}}\left(\alpha ,\;\beta ,\;\gamma ,\;b\right)=N_{\alpha }N_{\beta } \end{array}$$ (8)

where *N*_*α*_ and *N*_*β*_ are the number of *α*- and *β*-spin electrons in the outgoing projectile calculated from their respective electron densities $\rho_{\alpha }^{out.\;proj.}\left(r\right)$ and $\rho_{\beta }^{out.\;proj.}\left(r\right)$:

$$N_{\alpha }=\int \rho_{\alpha }^{out.\;proj.}\left(r\right)\;dr;\;N_{\beta }=\int \rho_{\beta }^{out.\;proj.}\left(r\right)\;dr$$ (9)

Eqs 8 and 9 are evaluated at the final simulation time, when the outgoing projectile and the clusters are well separated by at least 30.00 a.u. of length; therefore, *N*_*α*_ and *N*_*β*_ are the number of electrons unequivocally assigned to the distant outgoing projectile. Moreover, at those separations, *N*_*α*_ and *N*_*β*_ from Eq 9 and those from any ordinary electron population analyses (Mulliken, Löwdin, etc.) [53] become identical; this assures that *N*_*α*_ and *N*_*β*_ are free of any arbitrary criterion for electrons’ distributions over the projectile and clusters. Other calculated properties are the orientation-averaged 1-ET probabilities ${\bar{P}}_{1-\text{ET}}\left(b\right)$, ${\bar{P}}_{1-\text{ET}}\left(b\right)={\left(8\pi^{2}\right)}^{-1}\int_{0}^{2\pi }\int_{0}^{\pi }\int_{0}^{2\pi }P_{1-\text{ET}}\;\left(\alpha ,\;\beta ,\;\gamma ,\;b\right)\;\sin\beta$, and their *b* – weighted counterparts, $b{\bar{P}}_{1-\text{ET}}\left(b\right)$, where $\sigma_{1\text{-ET}}=2\pi \int_{0}^{\infty }b\;{\bar{P}}_{1-\text{ET}}\;\left(b\right)\;db$. ${\bar{P}}_{1-\text{ET}}\left(b\right)$ and $b{\bar{P}}_{1-\text{ET}}\left(b\right)$ reveal more mechanistic details than *σ*_1-*ET*_.

## Results and discussion

### Electron-transfer properties

The first property calculated in this investigation is the cluster-to-proton total 1-ET ICS, *σ*_1−*ET*_, for the H^+^ + (H_2_O)_n_ systems, *n* = 1–6, at *E*_*Lab*_ = 100 keV at the SLEND/6-31G* (*n* = 1–6) and SLEND/6-31G** (*n* = 1–5) levels. Both SLEND *σ*_1−*ET*_ for the monomeric system (*n* = 1) are listed in Table 2 along with their available counterparts from four experiments denoted as Exp. A to D [70–73], respectively. In addition, Table 2 includes results from two alternative theories, namely, Theory A: the basis generator method [23] (BGM), and Theory B: the continuum distorted wave-eikonal initial state (CDW-EIS) approximation [22]. From Table 2, one finds that the average experimental ICS, ${\bar{\sigma }}_{1-ET}^{Exp.}$, and its average relative error, *e*_*Exp*._, are 1.27 Å^2^ and ± 10.62%, respectively. The theoretical $\sigma_{1-ET}^{Theo.}$ ‘s and their average relative deviations ${\bar{\Delta }}_{Theo.}$ from the experimental values are: 1.54 Å^2^ and +21.8% for SLEND/6-31G*, 1.00 Å^2^ and +21.0% for BGM, and 0.589 Å^2^ and -53.4% for CDW-EIS. Only the BGM result is within the error bars of one experiment, Exp. D [73], with Δ_*Theo*._ = 11.5%, but it lies on the lowest fringe part of the error bar range. The SLEND/6-31G* result is very close to the result from Exp. C [72] with Δ_*Theo*._ = 11.6% and not far from entering the upper part of the error bar range. In absolute quantitative terms, the BGM and SLEND/6-31G* results are at the same level of accuracy and their agreement with the experimental data should be deemed satisfactory given the difficulty to both measure and predict the present 1-ET processes. Deviations of the obtained magnitude are not uncommon in measurements and predictions of similar complex processes (cf. Ref.[27] for the case of one experiment and four different theories including SLEND, where even higher deviations are observed). The CDW-EIS result compares less favorably with the experimental ones, being roughly a half of its experimental counterparts (${\bar{\Delta }}_{Theo.}$ = -53.4%). The SLEND/6-31** result also compares less favorably with the experimental ones, but, opposite to CDW-EIS, its value is roughly twice as much as the experimental one (${\bar{\Delta }}_{Theo.}$ = 63.0%). The reason and remediation of the SLEND/6-31** *σ*_1−*ET*_ overestimation will be discussed in detail in the Further Analysis Sub-section. It suffices to say here that this overestimation results from the *σ*_1−*ET*_ contamination with electron transfers to the continuum of unbound states.

**Table 2:** Water-to-proton 1-electron-transfer total integral cross sections (ICSs) *σ*_1−*ET*_ for H^+^ + H_2_O at *E*_*Lab*_ = 100 keV from experiments, SLEND theory and alternative theories: basis generator method (BGM) and continuum distorted wave-eikonal initial state (CDW-EIS) approximation.

**Experiment**	**ICS (Å**^**2**^**)**	**Exp. Error: Absolute (Relative)**
Exp. A: Rudd et al. [70]	1.23	∼ ± 0.123 (10.0%)
Exp. B: Gobet et al. (2001) [71]	1.35	
Exp. C: Gobet et al. (2004) [72]	1.38	± 0.094 (6.8%)
Exp. D: Luna et al. [73]	1.13	± 0.17 (15.0%)
**Theory**	**ICS (Å**^**2**^**)**	**Error: Absolute (Relative)**
SLEND/6-31G*	1.54	± 0.005 (0.3%)
SLEND/6-31G**	2.06	± 0.005 (0.2%)
Theory A: BGM, Murakami et al. [23]	1.00	
Theory B: CDW-EIS, Champion et al.[22]	0.589	

**Fig 3:**
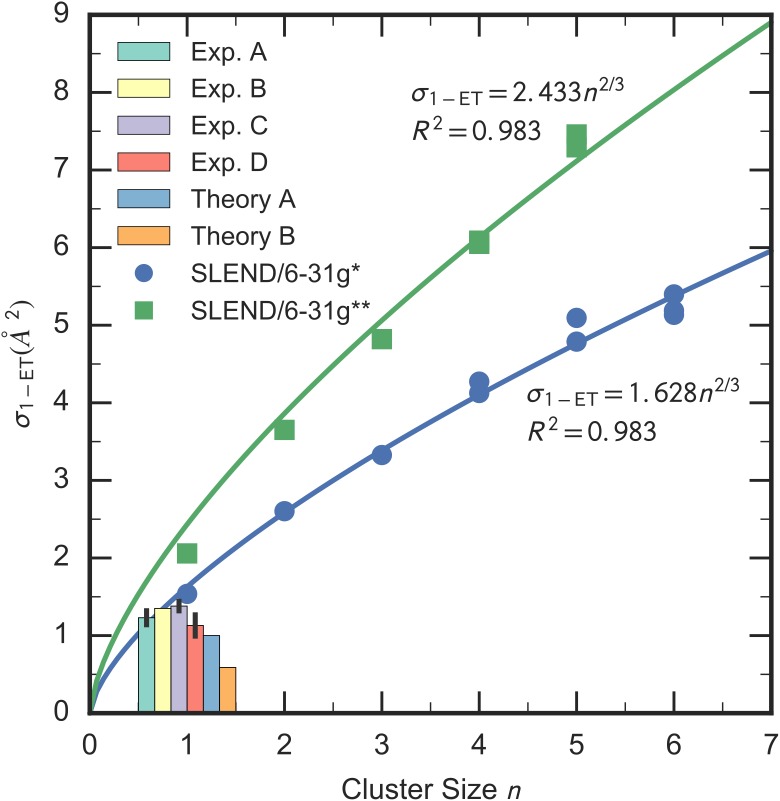
SLEND/6-31G* and /6-31G** target-to-proton total 1-ET ICSs *σ*_1−*ET*_ for H^+^ + (H_2_O)_1-6_ vs. the water cluster size *n*. Current data are in comparison with available experimental and theoretical *σ*_1−*ET*_ for *n* = 1 [Exp.: A [70], B [71], C [72] and D [73], Theory A: basis generator method (BGM) [23], Theory B: continuum distorted wave-eikonal initial state (CDW-EIS) approximation [22]]. SLEND values are fit to the scaling formula *σ*_1−*ET*_ (*x*) = *cn*^2/3^. The error of the SLEND ICSs is ± 0.005 Ä. The errors from the Theory A and B results compared herein were not reported.

The SLEND *σ*_1−*ET*_ for the polymeric systems (*n* = 2–6) are listed in Table 3. Unfortunately, to the best of our knowledge, there are no alternative experimental or theoretical ICSs for H^+^ + (H_2_O)_2-6_; therefore, current SLEND *σ*_1−*ET*_ for these systems are predictive. To facilitate the comparison among all the considered *σ*_1−*ET*_, we plot them as a function of the cluster size *n* in Fig 3. There, each set of SLEND/6-31G* and /6-31G** *σ*_1−*ET*_ is fit to the scaling formulae *σ*_1−*ET*_ (*n*) = *cn*^2/3^, where *c* are fitting coefficients reported in Fig 3. These formulae are by no means arbitrary because they can be justified on physical grounds as follows. The volume *V*(*n*) of the (H_2_O)_*n*_ clusters should be approximately proportional to their size *n*, *V*(*n*) ∝ *n*. If the clusters are represented by the minimal spheres enclosing all their atoms, then their volume *V*(*n*) and external area *A*(*n*) are $V\;\left(n\right)=\left(4/3\right)\;\pi \;R_{n}^{3}$ and $A\left(n\right)=4\pi \;R_{n}^{2}$, respectively, where *R*_*n*_ is the radius of the (H_2_O)_*n*_ sphere; therefore, *A*(*n*) ∝ *V* (*n*)^2/3^ ∝ *n*^2/3^. In turn, the *σ*_1−*ET*_ are proportional to the effective external area *A*(*n*) of the (H_2_O)_*n*_ exposed to the incident H^+^ for ET processes [42]; therefore, *σ*_1−*ET*_ ∝ *A*(*n*) ∝ *n*^2/3^ ⇒ *σ*_1−*ET*_ (*n*) = *cn*^2/3^. In relative quantitative terms, the SLEND/6-31G* and /6-31G** *σ*_1−*ET*_ fit remarkably well into the physically justified formulae *σ*_1−*ET*_ (*n*) = *cn*^2/3^ with correlation factors *R*^2^ = 0.983 in both cases (cf. Fig 3). This indicates that regardless of their absolute quantitative performance, the SLEND *σ*_1−*ET*_ scale correctly with the number of water molecules in the clusters. Therefore, with these fitting formulae, one can estimate the *σ*_1−*ET*_ of the immediately larger clusters: e.g., $\sigma_{1-ET}^{6-31G^{*}}$ = 5.96 and 6.51 Å for *n* = 7 and 8, respectively; these estimated values should be interpreted as average *σ*_1−*ET*_ over the various (H_2_O)_7_ and (H_2_O)_8_ [34] isomers, respectively. Inspection of Tables 2 and 3 and Fig 3 reveals that the SLEND/6-31G** *σ*_1−*ET*_ are always higher in value than the SLEND/6-31G* ones for each cluster as was the case with the H_2_O monomer. Inspection of Table 3 and Fig 3 reveals that the SLEND *σ*_1−*ET*_ for the various isomers appearing at a given *n* ≥ 4 do not significantly differ in their values; this implies that these *σ*_1−*ET*_ are rather insensitive to the varying isomers’ structures as targets. A similar finding was observed in the *σ*_1−*ET*_ of H^+^ + DNA/RNA bases at *E*_*Lab*_ = 80 keV [27], where similar bases differing in their structure even more than isomers exhibited close values of *σ*_1−*ET*_. We expected that various *σ*_1−*ET*_ (*n*) = *cn*^2/3^ formulae differing in their coefficient *c* would exclusively connect values from different sets of clusters—e.g. a single *σ*_1−*ET*_ (*n*) = *cn*^2/3^ formula might have only fit well with results from the quasi-planar clusters (monomer, dimer, trimer, tetramer a, pentamer a and hexamer c), another single formula with other type of clusters, etc., but that is not case. In fact, the uniformity among the SLEND *σ*_1−*ET*_ (*n*) values for isomers at a given *n* led us to fit all of them with a single formula per basis set. Furthermore, we expected that the *σ*_1−*ET*_ of the drop-like prism and cage (H_2_O)_6_ isomers would differ sharply from the *σ*_1−*ET*_ of the quasi-planar/multiplanar isomers so that it would be impossible to fit drop-like and non-drop-like *σ*_1−*ET*_ with a single formulae. Such a hypothetical fitting failure would manifest as a “phase transition” discontinuity from non-drop-like to drop-like *σ*_1−*ET*_. However, no such “phase transition” is observed in the selected series of (H_2_O)_1-6_ isomers. Thus, unlike the case of structural and solubility properties [35–37], the “magic number” of six waters in the prism and cage (H_2_O)_6_ isomers do not bring about any hint of water radiolysis processes in bulk water. Likely, an extension of the current (H_2_O)_1-6_ series with the (H_2_O)_7-20_ isomers may bring about some type of bulk-water manifestations.

**Table 3:** SLEND cluster-to-proton 1-electron-transfer integral cross sections *σ*_1−*ET*_ for H^+^ + (H_2_O)_*n*_, *n* = 2–6, at *E*_*Lab*_ = 100 keV. Cf. Fig 1 for the structures of the water cluster isomers. The error of the SLEND integral cross sections is ± 0.005 Ä.

Water Cluster	Basis Set	1-electron ICS (Å^2^)
Dimer	6-31G*	2.60
Dimer	6-31G**	3.65
Trimer	6-31G*	3.33
Trimer	6-31G**	4.82
Tetramer a	6-31G*	4.13
Tetramer a	6-31G**	6.05
Tetramer b	6-31G*	4.27
Tetramer b	6-31G**	6.09
Pentamer a	6-31G*	5.09
Pentamer a	6-31G**	7.46
Pentamer b	6-31G*	4.79
Pentamer b	6-31G**	7.29
Hexamer a (prism)	6-31G*	5.13
Hexamer b (cage)	6-31G*	5.19
Hexamer c	6-31G*	5.40

**Fig 4:**
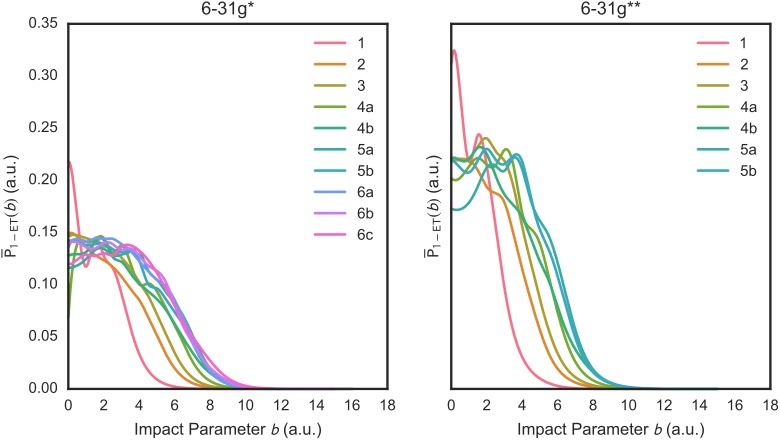
Orientation-averaged target-to-proton 1-ET probabilities ${\bar{P}}_{1-ET}^{}(b)$ at the SLEND/6-31G* (left panel) and /6-31G** (right panel) levels vs. the impact parameter *b* for the investigated (H_2_O)_1-6_. Water cluster isomers are denoted with the number code in Fig 1 (1, 2 … 6b, 6c).

**Fig 5:**
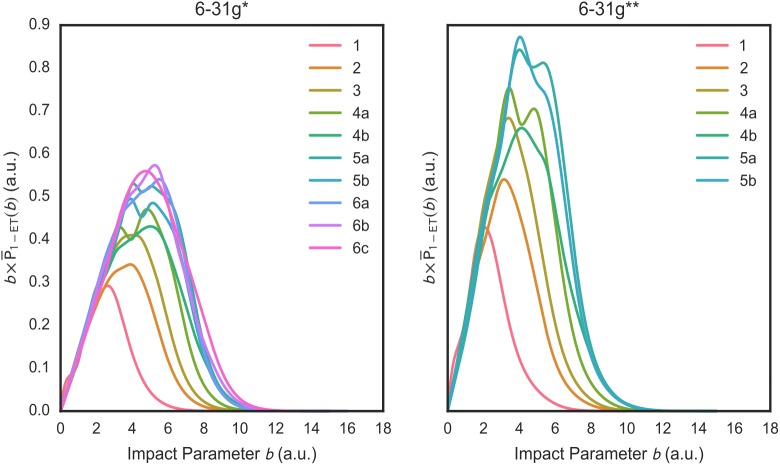
Orientation-averaged impact-parameter-weighted target-to-proton 1-ET probabilities $b{\bar{P}}_{1-ET}^{}(b)$ at the SLEND/6-31G* (left panel) and /6-31G** (right panel) levels vs. *b* for the investigated (H_2_O)_1-6_. Water cluster isomers are denoted with the number code in Fig 1 (1, 2 … 6b, 6c).

Total 1-ET ICSs *σ*_1−*ET*_ are not very detailed properties and cannot reveal some dynamical details of 1-ET processes. Therefore, Figs 4 and 5 show the orientation-averaged 1-ET probabilities ${\bar{P}}_{1-ET}^{}(b)$ and their *b* – weighted counterparts $b{\bar{P}}_{1-ET}^{}(b)$ vs. *b* for the considered (H_2_O)_1-6_ isomers. With both basis sets, the ${\bar{P}}_{1-ET}^{}(b)$ are high in value at small impact parameters (roughly, *b*≤6 a.u.) corresponding to close projectile-cluster encounters but they decrease rapidly at larger impact parameters. The ${\bar{P}}_{1-ET}^{}(b)$ vs. *b* plots show a variable number of maximum peaks (from one to three) depending on the considered water cluster. The $b{\bar{P}}_{1-ET}^{}(b)$ vs. *b* plots exhibit similar patterns to those of the ${\bar{P}}_{1-ET}^{}(b)$ but modulated by the *b* value. As the integrands of the *σ*_1−*ET*_, the $b{\bar{P}}_{1-ET}^{}(b)$ plots indicate that the most important contributions to the *σ*_1−*ET*_ come from 1-ET processes arising from intermediate impact parameters (roughly, 2 a.u. ≤ *b* ≤ 9 a.u.).

### Fragmentation reactions

**Fig 6:**
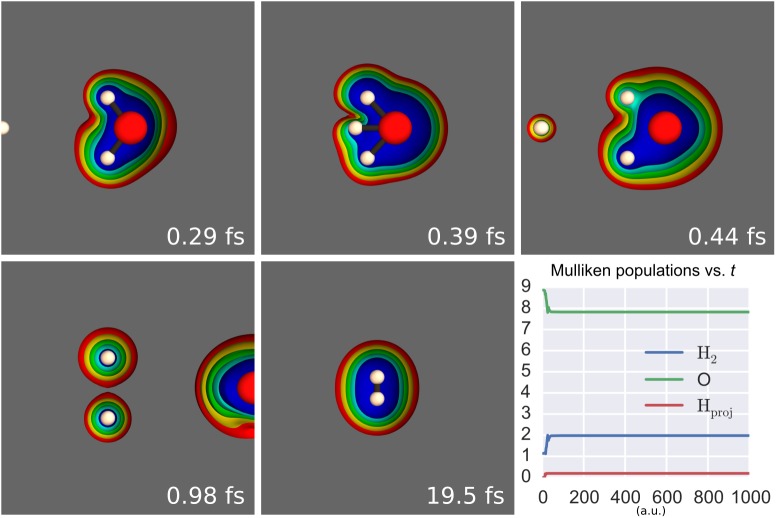
Example of a SLEND/6-31G* simulation of H^+^ + H_2_O leading H_2_O to fragment into H_2_ and O. Panels 1 to 5 are frames of this simulation animation, where colored spheres represent the nuclei (white = H and red = O) and colored clouds represent selected electron density iso-surfaces (from red = lowest density to blue = highest density). Panel 6 shows the Mulliken populations of the projectile H_*proj*._ and of the O and H_2_ moieties of/from H_2_O vs. time in a.u. Mulliken populations at final time indicate that ${\text{H}}_{proj}^{+}+{\text{H}}_{2}\text{O}\to {\text{H}}_{proj}^{q_{1}}+{\text{H}}_{2}^{q_{2}}+{\text{O}}_{}^{q_{3}}$ with *q*_1_ = 1−*N*_1_ = +1, *q*_2_ = 2−*N*_2_ = 0 and *q*_3_ = 8−*N*_3_ = 0.

Unlike time-independent scattering methods [21, 22, 74] applicable to PCT, SLEND simulations can reveal the reactants-to-products time evolution of the chemical reactions underlying the 1-ET processes. To study those reactions, the SLEND/6-31G* simulations to calculate *σ*_1−*ET*_ of H^+^ + (H_2_O)_1-4_ (only tetramer a for *n* = 4) were prolonged from their simulation times of 30.00 a.u. (0.7257 fs) to 1,000 a.u. (24.19 fs) using the impact parameter grids [*b*]_2_ in Table 1. This much longer simulation time allows for the manifestation of fragmentation reactions that occur at longer time scales. In fact, no fragmentation was observed within the original time of 30.00 a.u. As Eq 6 shows, the final SLEND electronic wavefunction |**z**(*t*),**R**(*t*)⟩ is a superposition of various UHF states corresponding to various products’ channels [69, 75], e.g. ${\text{H}}_{proj.}^{+}+{\text{H}}_{2}\text{O}\to {\text{H}}_{proj.}^{q_{1}=+1}+{\text{H}}_{2}^{q_{2}=0}+{\text{O}}_{}^{q_{3}=0}$ or $\to {\text{H}}_{proj.}^{q_{1}=0}+{\text{H}}_{2}^{q_{2}=+1}+{\text{O}}_{}^{q_{3}=0}$ or $\to {\text{H}}_{proj.}^{q_{1}=-1}+{\text{H}}_{2}^{q_{2}=+1}+{\text{O}}_{}^{q_{3}=+1}$, etc., where ${\text{H}}_{proj.}^{q_{1}=0,+1,-1}$ is the incoming/outgoing projectile; these channel states occur with different probabilities [69, 75]. Therefore, when Eq 9 is applied to all the well-separated fragments at final time, *N*_*α*_ and *N*_*β*_ and their corresponding charges *q*_*i*_ (e.g. *q*_1_ = 1−*N*_*α*_−*N*_*β*_ for the final ${\text{H}}_{proj.}^{q_{1}}$) are not necessarily integer numbers corresponding to canonical chemical species (e.g. ${\text{H}}_{proj}^{q_{1}=+1}$, ${\text{H}}_{proj}^{q_{1}=0}$ and ${\text{H}}_{proj}^{q_{1}=-1}$) but fractional numbers as the averages of the number of electrons and charges over the channels’ probabilities. This was always the case in all the present simulations not leading to clusters fragmentations (i.e. ${\text{H}}_{proj.}^{+1}+{\left({\text{H}}_{2}\text{O}\right)}_{n}\to {\text{H}}_{proj.}^{q_{1}}+{\left({\text{H}}_{2}\text{O}\right)}_{n}^{1-q_{1}}$, with *q*_*i*_ continuously varying in the range −1≤*q*_*i*_≤+1). However, as in previous SLEND studies of H^+^ + (H_2_O)_1–4_ at *E*_*Lab*_ = 1 keV [6], the present simulations leading to clusters fragmentations always bring about outgoing projectiles ${\text{H}}_{proj.}^{q_{1}}$ and clusters fragments ${\text{A}}^{q_{2}}$ with *q*_1_ = +1 and *q*_2_ = 0, respectively (cf. Fig 6 caption). Thus, the present SLEND simulations predict that the fragmentation channel leading to outgoing H^+^ and neutral fragments predominates over the others. However, it is known experimentally that proton-water collisions lead to fragmentations into ions [76–78]. SLEND can properly describe fragmentations into ions as shown in previous studies [e.g. cf. Refs. [79, 80]]. Therefore, to allow the manifestation of those types of fragmentations here, it will be necessary to prolong even further the simulation time of the present calculations or, more likely, increase the number of total simulations by using a finer grid. Such improvements entail further computational cost and will be attempted later.

The predicted fragmentations are:

**H**^**+**^ **+ H**_**2**_**O simulations:** 9 out of 252 simulations exhibited the H_2_O target fragmenting into: H + OH (2 simulations), H + H + O (6 simulations) and H_2_ + O (1 simulation, cf. Fig 6).

**Fig 7:**
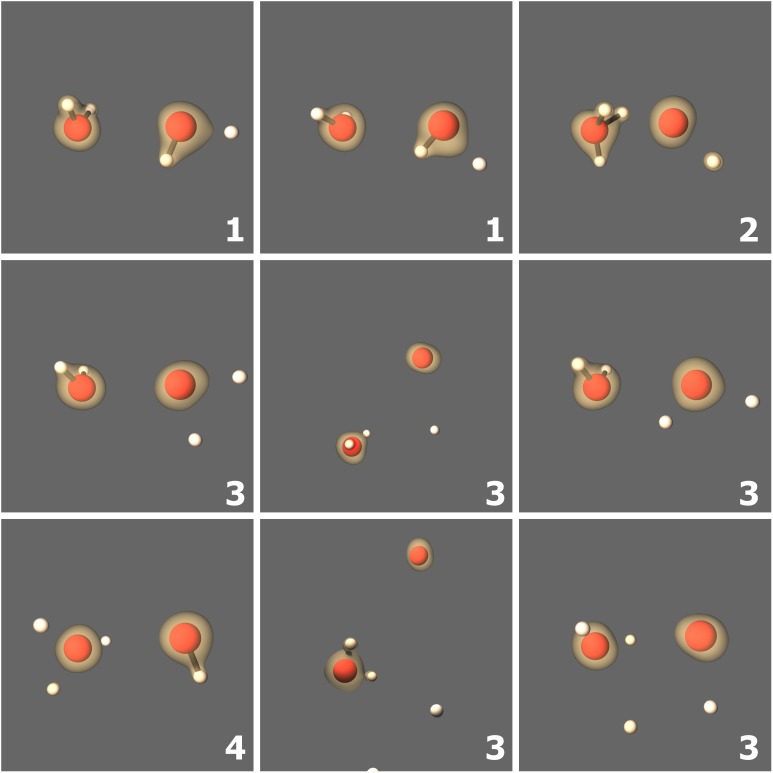
Target fragments from individual SLEND/6-31G* simulations of H^+^ + (H_2_O)_2._. Colored spheres represent the nuclei (white = H and red = O) and brown clouds represent selected electron density iso-surfaces. The predicted fragments from (H_2_O)_2_ are labeled and identified as 1: H_2_O + HO + H, 2: H_3_O + O + H, 3: H_2_O + 2H + O, and 4: H_3_O + OH.

**H**^**+**^ **+ (H**_**2**_**O)**_**2**_ **simulations:** 9 out of 540 simulations exhibited the (H_2_O)_2_ target fragmenting into: H_2_O + HO + H (2 simulations), H_3_O + O + H (1 simulation), H_2_O + 2H + O (5 simulations) and H_3_O + OH (1 simulation, cf. Fig 7).

**H**^**+**^ **+ (H**_**2**_**O)**_**3**_ **simulations:** 3 out of 540 simulations exhibited the (H_2_O)_3_ target fragmenting into: H_3_O + OH + H_2_O (2 simulations) and H + OH + 2H_2_O (1 simulation).

**H**^**+**^ **+ (H**_**2**_**O)**_**4**_ **(tetramer a) simulations:** 10 out of 748 simulations exhibited the (H_2_O)_4_ target fragmenting into: (H_2_O)_2_ + 2H_2_O (4 simulations), H_3_O + OH + 2H_2_O (4 simulations), an H + OH + 3H_2_O (2 simulations)

In conclusion, present SLEND/6-31G* simulations predict the DNA-damaging radicals H, OH, O and H_3_O, and the innocuous species H_2_O and (H_2_O)_2_ as water radiolysis products.

To illustrate the predicted fragmentations, we present a few animation stills from some representative simulations. Fig 6 shows five sequential stills of the animation of ${\text{H}}_{proj.}^{+}+{\text{H}}_{2}\text{O}\to {\text{H}}_{proj.}^{+}+{\text{H}}_{2}+\text{O}$ and a sixth panel plotting the Mulliken populations of the H_*proj*._, H_2_ and O moieties vs. time. This last panel permits the observation of the time evolution of the electrons. Mulliken populations are basis-set dependent and, more importantly, somewhat arbitrary in the way they distribute electrons over neighboring fragments. However, in previous SLEND studies [6, 75], Mulliken populations were good predictors for the time-evolution of the electrons over atoms and fragments. Furthermore, when all the fragments are well-separated at final time, the Mulliken populations converge to the unequivocal *N*_*α*_ and *N*_*β*_ in Eq 9 (cf. Fig 6 caption). In Fig 6, the colored spheres represent the classical nuclei (white = H and red = O), and the colored clouds represent selected electron density iso-surfaces (from red = lowest density to blue = highest density). The incoming projectile ${\text{H}}_{proj.}^{+}\;$ passes in between the H atoms of H_2_O, hits the O atom and bounces back. As a result of this collision, the H_2_ and O moieties of H_2_O break apart; the ejected H_2_ moiety undergoes a series of strong oscillations ranging from the near dissociation of H_2_ into H atoms to these atoms’ recombination back to H_2_. Finally, Fig 7 shows the nine predicted fragments in H^+^ + (H_2_O)_2_.

### Further analysis and improvements

SLEND/6-31G** *σ*_1−*ET*_ compares unsatisfactorily with experiments in contrast to SLEND/6-31G* *σ*_1−*ET*_. Indeed, it is surprising that SLEND/6-31G** performs worse than SLEND/6-31G* in these calculations since common knowledge dictates that the 6-31G** basis set is better than the 6-31G* one. 6-31G** is constructed from 6-31G* by augmenting the latter with p-type basis functions on the hydrogen atoms; thanks to this augmentation, 6-31G** provides better time-independent molecular properties than 6-31G*. For instance, the UHF/6-31G** energies and geometries of (H_2_O)_1–6_ are more accurate than the UHF/6-31G* ones. However, this comparative time-independent performance does *not* necessarily extend to time-dependent calculations since these basis sets were designed to calculate static properties. To explain the SLEND/6-31G** *σ*_1−*ET*_ overestimation, one should remember that the main component in the bound-to-bound SLEND *σ*_1−*ET*_ is the 1-ET probability *P*_1−ET_ (cf. Eqs 7 and 8). However, as derived in Ref. [69] and supposed in previous SLEND studies [29, 75], the *P*_*n*−ET_, 0 ≤ *n* ≤ 2, in Eq 8 assume that the probabilities of ETs from the target A to the projectile H^+^ are dominated by transitions with electrons transferring into the localized, discrete, bound states of H: H^+^ + A: → H⋅ + A⋅^+^ [pure charge-transfer (CT) processes]; instead, transitions with electrons scattering into the delocalized, continuous, unbound states of H are considered negligible: H^+^ + A: → (H^+^ + *e*^−^) + A⋅^+^ [direct ionization (DI) processes]. Typical quantum chemistry basis sets, such as 6-31G* and 6-31G**, are ultimately based on localized primitive Gaussian functions so that occupied spin-orbitals {*ψ*_*h*_} below the Fermi level represent localized, bound states in the discrete part of the spectrum. However, as a by-product of the UHF procedure, diffuse virtual spin-orbitals {*ψ*_*p*_} above the Fermi level approximately represent some of the delocalized, unbound states in the continuous part of the spectrum. Therefore, the virtual space constitutes the so-called quasi-continuum that may accommodate DI processes. However, if the basis set is not large, the DI contributions of a small quasi-continuum to the ET processes become negligible in comparison to the CT contributions of the occupied space. Under those conditions, the ET probabilities *P*_*n*−ET_ in Eq 8 basically correspond to bound-to-bound (occupied-space-to-occupied-space) CT processes. For that reason, with relatively small basis sets, those *P*_*n*−ET_ consistently rendered correct bound-to-bound CT *σ*_1−*ET*_ in various systems [26, 27, 29]. However, if the basis set is large, the DI contributions of an enlarged quasi-continuum may become substantial. If so, the *P*_*n*−ET_ in Eq 8 and resulting *σ*_*n*−*ET*_ no longer correspond to pure bound-to-bound CT processes since they get contaminated with bound-to-quasi-continuum DI contributions. Therefore, one can hypothesize that SLEND with the smaller 6-31G* basis set can predict genuine CT *σ*_*n*−*ET*_ via Eq 8 but not with the larger 6-31G** one.

To verify the above hypothesis, we performed a series of SLEND simulations on the simple model system: H^+^ + H at 40 keV ≤ *E*_*Lab*_ ≤ 100 keV with the 6–31^++^G** basis set. The latter produces the best DI results for H^+^ + H as shown shortly. However, instead of using *P*_1−*ET*_ in Eq 8 for *σ*_1−*ET*_, the final-time electronic wavefunction $\left|\Psi_{e}^{SLEND}\left(t_{f}\right)\right\rangle$, Eq 6, was projected onto the ground |0⟩ and excited states |… *h* → *p* …⟩ of the target and the projectile. In this way, the evaluation of ET probabilities could distinguish the cases with the electron transferring into bound or unbound states of H. In the 40 ≤ *E*_*Lab*_ ≤ 80 keV range, where experimental results are available [81], the DI ICSs, *σ*_*DI*_, deviate less than 10% from experimental data [81], with the best agreement at *E*_*Lab*_ = 60 keV: $\sigma_{DI}^{SLEND}$ = 13.9Å and $\sigma_{DI}^{EXPT.}$ = 13.8 Å ⇒ deviation Δ_*Theo*._ = 0.7%. Notably, these calculations produced accurate results even though ETFs were neglected as in Eq 4; this gives extra support to the ETFs’ neglect in the H^+^ + (H_2_O)_*n*_ simulations and ruled it out as a source of the SLEND/6-31G**σ_1−*ET*_ overestimation. For H^+^ + H at *E*_*Lab*_ = 100 keV, SLEND *σ*_*DI*_ is 0.92 Å^2^. If *P*_1−*ET*_ in Eq 8 is used to calculate CT ICSs, *σ*_*CT*_, a *σ*_*DI*_ contribution of 0.92 Å will be spuriously added to the *σ*_*CT*_ making it overestimated. If this DI contribution is assumed to be similar to that in H^+^ + H_2_O, the overestimated SLEND/6-31G** $\sigma_{CT}^{SLEND}$ via Eq 8 can be corrected by subtracting the $\sigma_{DI}^{SLEND}$ part from it: $\sigma_{CT}^{SLEND}$ = 2.06 Å2–0.92 Å^2^ = 1.14 Å^2^; this places SLEND/6-31G** $\sigma_{CT}^{SLEND}$ within the range of the four experimental values and closest to that from Exp. D [73]: $\sigma_{CT}^{Expt.D}$ = 1.13 Å ⇒ Δ_*Theo*._ = 0.8%. A similar correction might occur with the SLEND/6-31G* $\sigma_{CT}^{SLEND}$ but it will be far smaller due to a smaller virtual space as suggested by previous calculations with comparable basis sets [26, 27, 29]. The calculation of the CT *σ*_*CT*_ in H^+^ + H_2_O is more complicated than that of H^+^ + H because the former has more than one electron. For H^+^ + H_2_O, numerous excited states |… *h* → *p* …⟩ from |0⟩ forming a full CI expansion should be generated so that $\left|\Psi_{e}^{SLEND}\left(t_{f}\right)\right\rangle$ in Eq 6 can be projected on all those states. This more demanding capability is not currently available in PACE but is under development.

## Conclusions

To model microscopic processes in PCT [1–7], the SLEND method was applied to the H^+^ + (H_2_O)_*n*_ systems at *E*_*Lab*_ = 100 keV with the 6-31G* (*n* = 1–6) and 6-31G** (*n* = 1–5) basis sets. Ten (H_2_O)_1–6_ clusters were selected for this study: eight exhibit mono- and di-cyclic quasi-planar/multiplanar structures [33–35] and two others, the prism and cage (H_2_O)_6_ isomers, exhibit multi-cyclic, three-dimensional, drop-like structures [34, 35]. These “smallest-drop” clusters were purposely included in this study in an attempt to reproduce early manifestations of bulk-water properties in PCT. Short-time SLEND/6-31G* (*n* = 1–6) and /6-31G** (*n* = 1–5) simulations render cluster-to-projectile total 1-ET ICS, *σ*_1−*ET*_, and 1-ET probabilities. In absolute quantitative terms, SLEND/6-31G*σ_1−*ET*_ compares satisfactorily with alternative experimental [70–73] and theoretical[22, 23] results only available for *n* = 1, and exhibits almost the same accuracy of the best alternative theoretical result from BGM [23] calculations. SLEND/6-31G** overestimates *σ*_1−*ET*_ and a detail account about the cause and remediation of this effect was presented. In relative quantitative terms, both SLEND/6-31* and /6-31G** *σ*_1−*ET*_ precisely fit into physically justified scaling formulae *σ*_1−*ET*_ (*n*) = *cn*^2/3^ as a function of the cluster size *n*. Long-time SLEND/6-31G* (*n* = 1–4) simulations predict the formation of the DNA-damaging radicals H, OH, O and H_3_O. While “smallest-drop” isomers were included, no incipient manifestations of bulk-water PCT properties are observed. Therefore, to capture bulk-water effects, simulations with larger water clusters are currently underway.
